# Oestrogen-deficiency induces bone loss by modulating CD14^+^ monocyte and CD4^+^ T cell DR3 expression and serum TL1A levels

**DOI:** 10.1186/s12891-019-2704-z

**Published:** 2019-07-12

**Authors:** Fraser L. Collins, Michael D. Stone, Jane Turton, Laura R. McCabe, Eddie C. Y. Wang, Anwen S. Williams

**Affiliations:** 1 Division of Infection and Immunity, School of Medicine, Cardiff University, Cardiff, UK; 2 University Hospital Llandough, Cardiff & Vale University Health Board, Cardiff, UK; 3 Department of Physiology, Michigan State University, East Lansing, MI USA

**Keywords:** Death receptor 3, DR3, TNF-like protein 1A, TL1A, Menopause, Oestrogen-deficiency, Osteoporosis

## Abstract

**Background:**

Oestrogen-deficiency induced by menopause is associated with reduced bone density and primary osteoporosis, resulting in an increased risk of fracture. While the exact etiology of menopause-induced primary osteoporotic bone loss is not fully known, members of the tumour necrosis factor super family (TNFSF) are known to play a role. Recent studies have revealed that the TNFSF members death receptor 3 (DR3) and one of its ligands, TNF-like protein 1A (TL1A) have a key role in secondary osteoporosis; enhancing CD14^+^ peripheral blood mononuclear cell (PBMC) osteoclast formation and bone resorption. Whether DR3 and TL1A contribute towards bone loss in menopause-induced primary osteoporosis however, remains unknown.

**Methods:**

To investigate this we performed flow cytometry analysis of DR3 expression on CD14^+^ PBMCs isolated from pre- and early post-menopausal females and late post-menopausal osteoporotic patients. Serum levels of TL1A, CCL3 and total MMP-9 were measured by ELISA. In vitro osteoclast differentiation assays were performed to determine CD14^+^ monocyte osteoclastogenic potential. In addition, splenic CD4^+^ T cell DR3 expression was investigated 1 week and 8 weeks post-surgery, using the murine ovariectomy model.

**Results:**

In contrast to pre-menopausal females, CD14^+^ monocytes isolated from post-menopausal females were unable to induce DR3 expression. Serum TL1A levels were decreased approx. 2-fold in early post-menopausal females compared to pre-menopausal controls and post-menopausal osteoporotic females; no difference was observed between pre-menopausal and late post-menopausal osteoporotic females. Analysis of in vitro CD14^+^ monocyte osteoclastogenic potential revealed no significant difference between the post-menopausal and post-menopausal osteoporotic cohorts. Interestingly, in the murine ovariectomy model splenic CD4^+^ T cell DR3 expression was significantly increased at 1 week but not 8 weeks post-surgery when compared to the sham control.

**Conclusion:**

Our results reveals for the first time that loss of oestrogen has a significant effect on DR3; decreasing expression on CD14^+^ monocytes and increasing expression on CD4^+^ T cells. These data suggest that while oestrogen-deficiency induced changes in DR3 expression do not affect late post-menopausal bone loss they could potentially have an indirect role in early menopausal bone loss through the modulation of T cell activity.

## Background

Osteoporosis is characterized by micro-architectural deterioration of bone tissue and low bone mass that consequently results in increased bone fragility and susceptibility to fracture [1]. In the United Kingdom more than 3 million people are estimated to have osteoporosis with 500,000 osteoporotic fractures every year; costing an estimated £1.8 billion in 2000 with a potential increase to £2.2 billion by 2025 [2, 3]. Osteoporosis can be characterized into two main forms: primary osteoporosis which occurs as part of aging and secondary osteoporosis, when bone loss is driven by a medical condition / disease or treatment [4]. The onset of menopause in females is a major factor in the development of primary osteoporosis. Loss of oestrogen results in two stages of bone loss: an early rapid loss of trabecular and cortical bone due to increased osteoclast activity and decreased osteoclast apoptosis, and a second slower prolonged loss due to decreased osteoblast activity [4, 5]. In contrast, bone loss due to secondary osteoporosis is caused by factors including but not limited to hyperparathyroidism, inflammatory bowel disease (IBD), type 1 diabetes (T1D), arthritis and glucocorticoid treatment. Several mechanisms are known to contribute to the pathology of menopause-induced primary osteoporosis such as increased expression of tumour necrosis factor (TNF) superfamily members TNFα (TNFSF2) and receptor activator of nuclear factor kappa-B ligand (RANKL; TNFSF11) [6–11]. It is currently unknown however, what role other members of the TNFSF play in this pathological bone loss. This study focused on the TNFSF members TNF-like protein 1A (TL1A, TNFSF15) and its only confirmed trans-membrane receptor, death receptor 3 (DR3; TNFRSF25) [12].

DR3 and its ligand TL1A have been implicated in the pathogenesis of numerous inflammatory conditions associated with secondary osteoporosis including: IBD and rheumatoid arthritis (RA) [13–15]. While the majority of DR3’s function has been attributed to its expression on T cells and the ability of TL1A to drive the proliferation of effector T cell subsets [16, 17], studies have identified expression of DR3 on the surface of osteoclast precursors and osteoblasts [14, 18, 19]. In vitro studies using circulating CD14^+^ monocytes, osteoclast precursors which migrate to bone to undergo differentiation into osteoclasts [20], identified cell surface expression of DR3 and that addition of TL1A to these cells significantly enhanced osteoclast proliferation and resorptive activity; increasing expression of the chemokine CCL3 and the enzyme matrix metallopepetidase 9 (MMP9) [14]. Furthermore, expression of DR3 has also been identified on human osteoblasts (OB) where, in vitro, it mediated apoptosis under narrowly regulated conditions [21]. These results suggest that signalling by TL1A through DR3 on CD14^+^ osteoclast precursors and osteoblasts could have an important direct role in the pathogenesis of menopause-induced primary osteoporosis; increasing bone resorption and decreasing bone formation. This is further supported by in vivo studies in the murine collagen-induced arthritis (CIA) model where ablation of DR3 was shown to protect against secondary osteoporosis at sites distal from the small joints [14]. Furthermore, CD4^+^ T cells have been identified as being significant drivers of bone loss following oestrogen deficiency [22], leading to the possibility that changes in DR3 / TL1A expression on these cells could potentially have an indirect effect on bone loss.

While the current data suggests that DR3 and TL1A are implicated in adverse bone loss associated with secondary osteoporosis, their complicity in the pathology of menopause-induced primary osteoporosis remains unknown. In the present study we investigated serum levels of TL1A and the expression of DR3 on peripheral blood CD14^+^ cells isolated from pre-menopausal, post-menopausal and late post-menopausal osteoporotic females to determine whether changes in oestrogen status result in significant modulation of these TNFSF members. We demonstrate for the first time that, in contrast to pre-menopausal females, post-menopausal serum levels of TL1A are not significantly elevated and that DR3 expression is not induced on CD14^+^ monocytes. However, utilizing the murine ovariectomy (OVX) model of oestrogen-deficiency we reveal that early post-OVX expression of DR3 on CD4^+^ T cells is significantly elevated suggesting that DR3 and TL1A could play a potentially indirect role in early post-menopausal bone loss.

## Methods

### Ethical approval

Ethical approval for the isolation of patient blood was obtained from the South East Wales Research Ethics Committee (REC reference number: 10/WSE02/44). Patients were recruited to one of two cohorts: postmenopausal (*n* = 2) and postmenopausal osteoporotic with fracture (*n* = 4). Inclusion and exclusion criteria are outlined in Table 1. Patients with a t score lower than − 2.5 were deemed to be osteoporotic. Pre-menopausal control (*n* = 6) and additional post-menopausal control (*n* = 4) blood samples were obtained in-house with ethical approval provided by the Medical / Dental School Research Ethics Committee (MDSREC Reference Number: 09/21).

**Table 1:** Study Criteria

Inclusion Criteria	Exclusion Criteria
Attending for first bone density scan	Recent fracture (in the past 3 months)
Adult	History of corticosteroid use (except inhaled or topical)
Female	Anti-TNF therapy
Post-Menopausal	Patients with known seropositive rheumatoid arthritis, inflammatory bowel disease
	Recent history of continuous treatment with bisphosphonate, calcitonin, Strontium ranelate or parathyroid hormone (i.e. for more than 3 months)
	Patients with known primary hyperparathyroidism
	Currently taking part in another study

### Flow cytometry

Blood samples were collected in heparin coated tubes and processed within 2 h. Peripheral blood mononuclear cells (PBMCs) were isolated (pre-menopausal *n* = 6, post-menopausal *n* = 6 and osteoporotic *n* = 4) by density gradient centrifugation using Histopaque-1077 (Sigma), and CD14^+^ monocytes isolated by magnetic cell sorting following manufacturer’s instructions (Miltenyi Biotec, UK). Cells were stained in phosphate buffered saline (PBS) containing 1% (v/v) foetal calf serum (FCS) with anti-DR3-PE (clone JD3) and anti-CD14-FITC (clone 61D3) (eBioscience) for 30 min at 4 °C. Data were acquired on an Accuri C6 flow cytometer and analysed with FlowJo V10.

For murine splenic T cell analysis, spleens (*n* = 4–8) were isolated and homogenized into a single cell suspension. Red blood cells were removed with 1x RBC lysis buffer (eBioscience) according to manufacturer’s instructions. 1 × 10^6^ cells were incubated with Fc block (BD Pharmingen, CA, USA) for 15 min. Cells were stained with anti-mouse CD4-eF450 (RM 4–5, eBioscience) and anti-mouse DR3-PE (4C12, BioLegend) for 30 min at 4 °C. Data were acquired on a BD LSRII (Becton Dickinson, Franklin Lakes, NJ) and analysed with FlowJo (Version 10; FlowJo, LLC, Ashland OR).

### Osteoclastogenesis assays

Isolated CD14^+^ PBMCs (6.4 × 10^4^) were added to ivory discs in RPMI supplemented with 10% FCS, 20 mM L-glutamine and 50 μg/ml Penicillin/Streptomycin (RPMI-10). After 2 h at 37 °C 5% CO_2_, discs were transferred to 48-well plates and RPMI-10 with macrophage colony stimulating factor (MCSF; 5 ng/ml) added. Media was replenished after 3 days and cells stained for DR3 after 7 days (classified as day 0 for OC assays). Media was replenished every 3–4 days using RPMI-10 supplemented with MCSF (5 ng/ml), RANKL (5 ng/ml) and anti-polyHistidine (2.5 μg/ml) all from R&D systems. Supernatants were stored at − 80 °C for further analysis. Two discs per condition were stained for tartrate resistant acid phosphatase (TRAP) on day 14. Images of five random areas of the discs were taken at × 10 magnification using a BX41 microscope and Camedia C-3030 camera (Olympus, UK) and cropped to represent 1000 μm^2^ (Corel Paint Shop Pro, Corel, UK). The number of TRAP-positive multinucleated cells and TRAP-negative/positive mononucleated cells were counted and results reported per disc.

### Patient serum analysis

Serum levels of TL1A (Peprotech, London, UK), the chemokine CCL3 and Total matrix metalloproteinase (MMP)-9 (R&DSystems, Abingdon, UK) were measured by ELISA according to manufacturer’s instructions.

### Ovariectomy (OVX) surgeries

Female BALB/c mice, 11 weeks of age, were obtained from The Jackson Laboratory (Bar Harbour, Maine). Mice were allowed to acclimatize to the specific pathogen free animal facility for 1 week prior to start of experiment. Animals were randomly split into two groups: sham control or OVX (*n* = 4–8). For sham and OVX surgeries, mice were anesthetized with isofluorane and a 2 cm lower mid-dorsal incision was made extending through the skin and muscle layers. Ovaries were isolated in both sham and OVX groups; ovaries were removed from the OVX cohort and incision sites closed using surgical staples in both sham and OVX mice. Mice were assessed daily for welfare and surgical staples removed 7 days post-surgery. Mice were housed in shoebox cages with environmental enrichment in groups of 4, provided with Teklad 2019 chow (Madison, WI) and water ad libitum and maintained on a 12-h light/dark cycle; all animal caretaking was performed by Michigan State University campus animal resources personnel. Mice were sacrificed in the morning 1 and 8 weeks post-surgery by overdose of inhalation anaesthetic followed by cervical dislocation. All animal procedures were approved by the Michigan State University Institutional Animal Care and Use Committee and conformed to NIH guidelines.

### Statistical analysis

All measurements are presented as the mean ± SEM. Unpaired t-test and 1-way ANOVA with Bonferroni or Dunnett’s post-test were performed using GraphPad Prism software version 6 (GraphPad, San Diego, CA, USA). *p*-values of < 0.05 were considered significant and *p*-values of < 0.01 highly significant.

## Results

### Patient bone parameters

Patient bone parameters were analysed by dual-energy X-ray absorptiometry (DXA) at three sites: hip, spine and neck of femur (Table 2). Bone mineral density (BMD) was not significantly different between the patient cohorts at the hip and spine. A significant decrease in neck of femur BMD was observed in the osteoporosis cohort when compared to the post-menopausal control (*p* < 0.01).

**Table 2:** Characterisation of patients included in study

	Pre-Menopausal	Post-Menopausal	Osteoporotic
Age (years)	21–32	54–58	68 ± 3
Hip BMD	–	0.72 ± 0.08	0.68 ± 0.03
Spine BMD	–	0.90 ± 0.06	0.83 ± 0.04
Neck of Femur BMD	–	0.76 ± 0.03	0.55 ± 0.01^b^
Weight (kg)	–	85.95 ± 11.65	62.73 ± 1.59
Serum TL1A (pg/ml)	123.5 ± 24.1	64.4 ± 22.9	117.3 ± 10.7
Serum CCL3 (pg/ml)	195.1 ± 88.3	82.6 ± 51.4	41.6 ± 30.4
Serum Total MMP-9 (ng/ml)	4.43 ± 0.2	5.12 ± 0.4	5.49 ± 0.1^a^

^a^Significantly different to pre-menopausal; ^b^Significantly different to post-menopausal

### Serum levels of TL1A, CCL3 and Total MMP-9

In autoimmune conditions such as rheumatoid arthritis levels of serum TL1A are significantly increased [23]. Levels were measured in patient serum to determine whether TL1A expression is a potential confounding factor in post-menopausal bone loss (Table 2). Interestingly, a 1.9-fold decrease in levels of circulating TL1A was measured in post-menopausal controls compared to the pre-menopausal controls, though this difference wasn’t significant. However, serum levels of TL1A in osteoporotic patients were comparable to the pre-menopausal control.

*Ex vivo* osteoclast cultures of cells isolated from pre-menopausal females revealed that TL1A induced expression of the chemokine CCL3 and MMP-9 [14]. As with TL1A, levels of serum CCL3 were decreased (2.4-fold) in the post-menopausal controls compared to pre-menopausal controls. Additionally, a 4.7-fold decrease was observed in the osteoporotic patients. In contrast to TL1A and CCL3 expression, levels of total MMP-9 were comparable in the post-menopausal controls but significantly increased in the osteoporotic patient serum (*p* < 0.05).

### Expression of DR3 on circulating CD14^+^ PBMCs

**Fig. 1:**
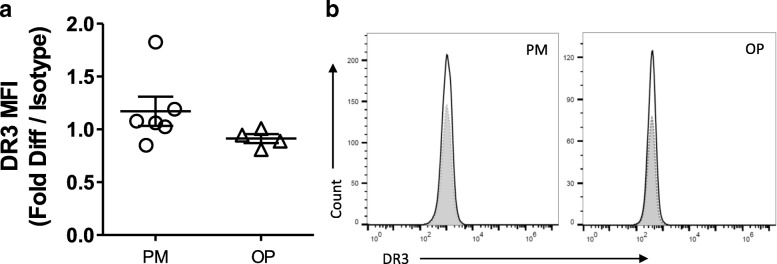
Expression of DR3 on Freshly Isolated Post-menopausal Human CD14^+^ Monocyte Osteoclast Precursors. CD14^+^ monocytes were isolated from post-menopausal females without (PM) (*n* = 6) and with osteoporosis (OP) (*n* = 4). DR3 expression was determined by flow cytometry. **a** DR3 expression was not detected on freshly isolated CD14^+^ monocytes. **b** representative histograms of DR3 expression. (Shaded peak = isotype; dark line = DR3 antibody)

To determine whether CD14^+^ osteoclast precursor DR3 expression is affected by menopause or osteoporosis, levels were analysed on freshly isolated CD14^+^ cells by flow cytometry (Fig. 1). No DR3 expression was detected on the surface of CD14^+^ monocytes isolated from the post-menopausal patients; comparable to that previously observed with cells isolated from pre-menopausal females [14].

**Fig. 2:**
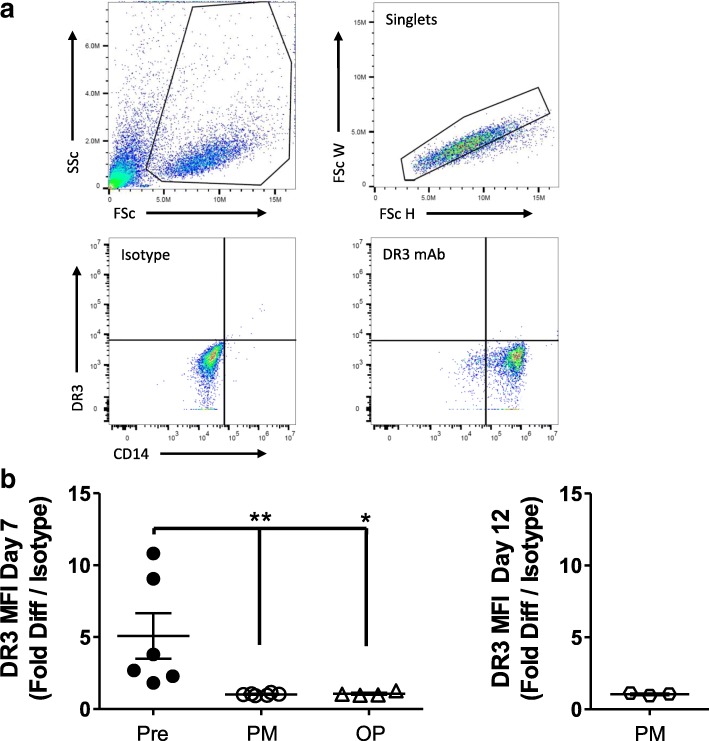
Expression of DR3 on Cultured Post-menopausal Human CD14^+^ Monocyte Osteoclast Precursors. CD14^+^ monocytes were isolated from pre-menopausal (Pre) (*n* = 6) and post-menopausal females, without (PM) (*n* = 6) and with osteoporosis (OP) (*n* = 4). Cells were cultured on ivory discs for 7 days in media + MCSF. DR3 expression was determined by flow cytometry. **a** Gating strategy for detection of DR3. **b** Fold difference in CD14^+^ monocyte DR3 expression after 7 days and 12 days in culture. After 7 days pre-menopausal expression of DR3 was significantly elevated compared to the post-menopausal (*p* < 0.01) and post-menopausal with osteoporosis (*p* < 0.05) cultures. DR3 expression was not induced on CD14^+^ cells isolated from post-menopausal females after 12 days in culture. Statistical analysis performed by 1 way ANOVA with Dunnett’s multiple comparison test

In contrast to pre-menopausal females however, no upregulation of DR3 expression was observed on cells isolated from post-menopausal females following culture in the presence of MCSF on ivory discs for 7 days (Fig. 2). To determine whether the cells required increased culture time for DR3 upregulation the culture period was extended to 12 days in 3 of the post-menopausal control samples. As with the 7 day time point no increase in DR3 expression was observed.

### CD14^+^ Osteoclastogenesis assays

**Fig. 3:**
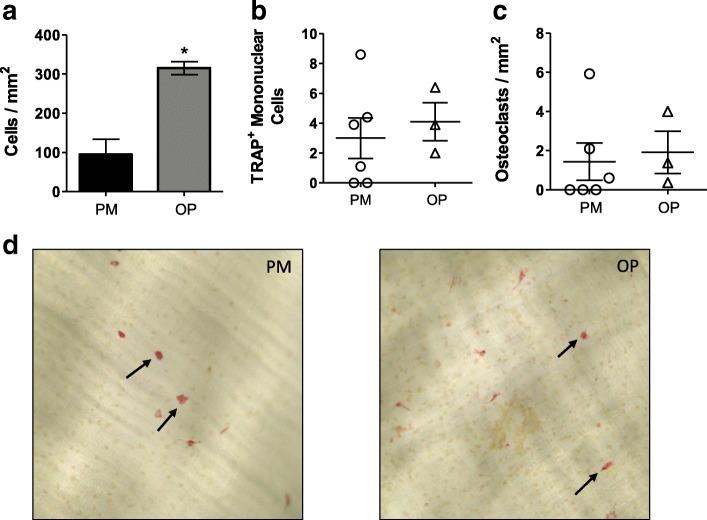
Osteoclastogenesis Assays CD14^+^ monocytes were isolated from post-menopausal females without (PM) (*n* = 6) and with osteoporosis (OP) (*n* = 4) and cultured on ivory discs for 7 days in media + MCSF. Cells were differentiated for 14 days in the presence of MCSF and RANKL. At experiment end- point cells were stained for TRAP. **a** Significantly increased cell number was observed in cultures derived from post-menopausal females with osteoporosis (*p* < 0.05). No difference was observed in the number of (**b**) TRAP^+^ mononuclear cells or (**c**) multinucleated osteoclasts. **d** Representative images of osteoclast cultures. Arrows indicate multinucleated TRAP^+^ osteoclasts. Statistical analysis performed by unpaired student’s t-test

Post-menopausal osteoporosis is characterized by a period of increased osteoclast activity resulting in significant bone loss [24]. We investigated, ex vivo, whether the lack of DR3 expression on post-menopausal CD14^+^ monocytes affected osteoclastogenesis. At the experiment endpoint (day 14), significantly elevated total cell numbers were observed in the osteoporotic patient-derived cultures compared to the post-menopausal controls (*p* < 0.05; Fig. 3a). Somewhat surprisingly however, no significant difference was observed between the cultures for TRAP^+^ mononuclear cells and TRAP^+^ multinucleated osteoclasts (Fig. 3b, c and d). Interestingly, the number of osteoclasts generated by the CD14^+^ cells isolated from the post-menopausal patients was approximately 3-fold lower than that previously reported from pre-menopausal females using the same system [14]. This suggests that while DR3 expression is not essential, its presence on precursors enhances osteoclastogenesis.

### CD4^+^ T Cell DR3 expression following loss of Oestrogen

**Fig. 4:**
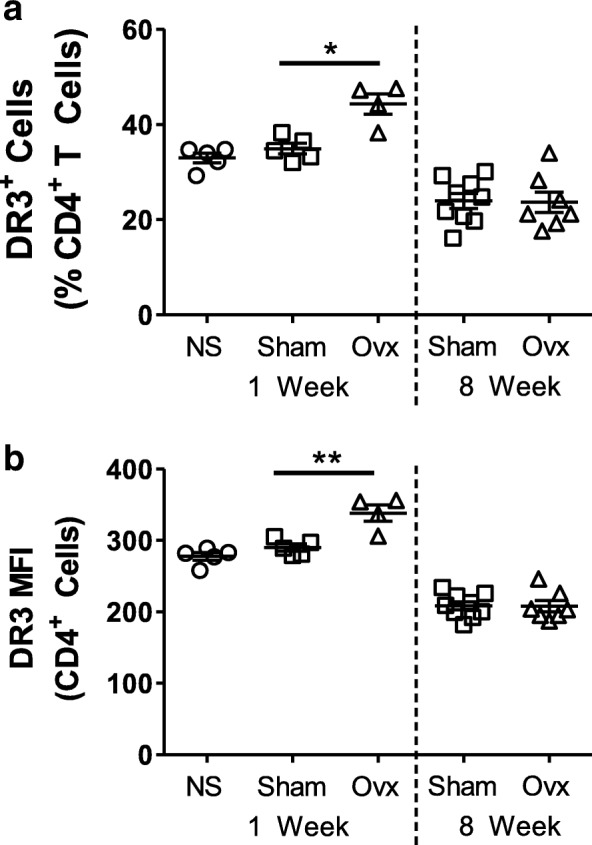
Expression of DR3 on Splenic CD4^+^ T Cells following Ovairectomy. Female BALB/c mice (12 weeks) underwent either sham or ovariectomy (OVX) surgery. The (**a**) number of DR3^+^ splenic CD4^+^ T cells and (**b**) DR3 expression (MFI) was analysed by flow cytometry 1 week and 8 weeks post-surgery. One week post-surgery significantly increased CD4^+^ DR3^+^ cell numbers (*p* < 0.05) and DR3 expression (*p* < 0.01) was observed in the OVX cohort compared to the sham cohort (*p* < 0.05). At 8 weeks post-surgery no difference was detected between the sham and OVX cohorts. Statistical analysis performed by unpaired student’s t-test

Previous studies have shown that osteoclast formation is increased in PBMC cultures isolated from women 1 year after menopause compared to women in menopause [7]. To determine whether DR3 expression could be playing a role in early post-menopausal PBMC osteoclast formation we utilized the murine ovariectomy (OVX) model of oestrogen-deficiency. Ovaries were removed from the mice and DR3 expression on splenic CD4^+^ T cells determined 1 week and 8 weeks post-surgery by flow cytometry (Fig. 4). The number of CD4^+^ DR3^+^ cells (*p* < 0.05) and DR3 expression (*p* < 0.01) was significantly increased in the OVX cohort 1 week post-surgery compared to the sham control. Eight weeks post-surgery however, no difference in DR3 expression was observed between the sham and OVX cohorts.

## Discussion

The pathology of osteoporosis is complex with many factors contributing to the increased bone loss and decreased bone formation. Of these factors, members of the TNFSF such as TNFα and RANKL are known to play critical roles. While roles for DR3 and its ligand TL1A have been demonstrated in numerous conditions that are associated with pathological bone loss [13–15], it is currently unknown whether DR3 and / or TL1A are affected by loss of oestrogen and contribute to the progression of menopause-induced primary osteoporosis. In the present study we reveal for the first time in humans that loss of oestrogen does not significantly affect serum levels of TL1A and that late post-menopause CD14^+^ monocyte osteoclast precursors are unable to induce DR3 expression. Furthermore, using the murine OVX model of oestrogen-deficiency, we demonstrate that early post-OVX DR3 expression is upregulated on CD4^+^ T cells but returns to normal levels at later time points, when compared to the sham control (mice that have the same surgery but whose ovaries remain intact). These data suggest that DR3 and TL1A could have a potential indirect role in increasing osteoclast formation and bone loss in the early stages of post-menopause.

Oestrogen deficiency associated with menopause has been linked to elevated levels of interleukin (IL)- 1β, IL-8 and TNFα in the serum [6] and increased expression of RANKL and TNFα in ex vivo cultures of PBMCs [7, 8], circulating monocytes [9] and bone marrow macrophages [10, 11]. In the present study, serum levels of TL1A were decreased in post-menopausal females but comparable in the osteoporotic cohort when compared to the pre-menopausal controls; suggesting that unlike TNFα, elevated expression of TL1A does not play a role in post-menopausal osteoporotic bone loss. However, it is important to note that the increased cytokine expression described in previous studies [6–11] were reported in samples isolated from early post-menopausal females. Interestingly, in the study by Bismar et al. [10] cytokine levels in late post-menopausal (70 ± 6 years old) bone marrow cultures were significantly lower than those from early post-menopausal women (51 ± 5 years old). These observations raise a couple of intriguing possibilities: firstly, that TL1A may be increased during the early stages of post-menopause when bone loss occurs but missed during this study and secondly; that due to physiological changes post-menopause, pre-menopausal levels of TL1A become pathological and contribute to bone loss. Having shown that serum levels of TL1A were not elevated in late post-menopausal osteoporotic patients we next investigated the effect on DR3 to determine if increased receptor expression could contribute to the increased bone loss associated with osteoporosis.

Previously we reported that circulating CD14^+^ monocytes, isolated from pre-menopausal females, express DR3 when cultured in the presence of MCSF on ivory discs and that treatment of these cells with TL1A resulted in significantly enhanced osteoclast formation [14]. Interestingly however, in the present study DR3 expression was not induced on CD14^+^ monocytes isolated from post-menopausal females, even when cultured for an extended period. The difference in DR3 expression between pre- and post-menopausal derived CD14^+^ monocytes suggests a critical change in these cells caused by menopause. The effects of aging and menopause on immune cell DR3 expression has not been extensively studied. In the only other study to investigate the effect of aging on DR3 expression, Slebioda et al. [25] noted differences in CD4^+^, CD8^+^ and CD20^+^ DR3 expression between healthy children (9.8 ± 4.3 years) and healthy adults (45.3 ± 10.5 years); suggesting that aging can have a significant effect on a cell’s DR3 phenotype. While in the Slebioda et al. [25] study aging did not affect CD14^+^ and CD11c^+^ DR3 expression, it does not rule out a change in DR3 phenotype on these cells due to menopause or as a person moves from adult (20–59 years) to elderly (60+ years); changes in monocyte and macrophage functions have been documented as impaired in aged animal models [26] and elderly individuals [27]. Furthermore, work by Sadeghi et al. [28] demonstrated that monocytic CD14 expression alters during aging; cells change from a CD14^bright^/CD16^dim^ phenotype in young individuals (30.5 ± 13.5 years) to a CD14^dim^/CD16^bright^ phenotype in the elderly (87.6 ± 14 years) demonstrating that these cells undergo a significant phenotypic change with age.

To determine whether the lack of DR3 expression on CD14^+^ monocytes affected osteoclast formation, we performed osteoclastogenesis assays. No difference in osteoclast potential was observed between the post-menopausal and osteoporotic cultures. However, levels of osteoclast formation were lower than we have previously reported for pre-menopausal controls under the same conditions [14]. This is in contrast to work published by D’Amelio et al. [7] who demonstrated significantly higher levels of ‘spontaneous’ osteoclast formation from osteoporotic patient PBMC cultures compared to pre-menopausal controls. The apparent discrepancy between the two studies however, could be explained by differences in methods used. In the D’Amelio et al. [7] study whole PBMC cultures were utilized as compared to CD14^+^ monocyte cultures used in this study. Increased production of pro- osteoclastogenic cytokines RANKL, MCSF and TNFα by PBMCs post-menopause has been demonstrated by a number of groups [7–9]. Elevated levels of these cytokines would have a significant effect on osteoclast formation in these cultures. Of the PBMCs, T cells are believed to play a particularly critical role in bone loss associated with oestrogen-deficiency [4]; loss of oestrogen has been observed to increase T cell expression of TNFα, RANKL and IL-17 [8, 29, 30]. Importantly, signalling by TL1A through DR3 on CD4^+^ T cells has been shown to induce expression of TNFα and IL-17 [17, 31, 32]; suggesting that changes in DR3 and TL1A signalling on these cells could indirectly affect osteoclast formation by modulating T cell cytokine expression. To investigate this we performed a murine model of ovariectomy and investigated splenic CD4^+^ T cell DR3 expression 1 and 8 weeks post-surgery as indicators of early and late post-menopause. Interestingly, DR3 expression on CD4^+^ T cells was significantly elevated at 1 week but not 8 weeks post-surgery. This raises the very significant possibility that elevated CD4^+^ T cell DR3 expression may contribute to the increased bone loss observed in early menopause.

In conclusion, we have demonstrated for the first time that loss of oestrogen has a significant effect on DR3 and TL1A expression. We reveal that post-menopause the ability to induce DR3 expression is lost from circulating CD14^+^ monocytes and serum TL1A levels trend downwards compared to pre-menopausal females. In contrast however, serum TL1A levels in post-menopausal osteoporotic females are comparable to pre-menopausal. However, due to the low numbers of patients recruited to the study, further work is required before the exact effect of menopause on DR3 and TL1A expression can be revealed. In addition, we further identify in a murine ovariectomy model of oestrogen loss that CD4^+^ T cell DR3 expression is significantly upregulated early on, suggesting that DR3 / TL1A signalling could indirectly contribute to the increased bone loss observed post-menopause through modulation of T cell activity.

## Data Availability

The datasets used and/or analysed during the current study are available from the corresponding author on reasonable request.

## Abbreviations

BMD: Bone mineral density
CIA: Collagen-induced arthritis
DR3: Death receptor 3
DXA: Dual-energy X-ray absorptiometry
IBD: Inflammatory bowel disease
MCSF: Macrophage colony stimulating factor
MMP-9: Matrix metalloproteinase 9
OVX: Ovariectomy
PBMCs: Peripheral blood mononuclear cells
PBS: Phosphate buffered saline
RA: Rheumatoid arthritis
RANKL: Receptor activator of nuclear factor kappa-B ligand
REC: Research Ethics Committee
T1D: Type 1 diabetes
TL1A: TNF-like protein 1A
TNF: Tumour necrosis factor
TNFSF: Tumour necrosis factor super family
TRAP: Tartrate resistant acid phosphatase

## References

[1] Various (1993). Consensus development conference: diagnosis, prophylaxis, and treatment of osteoporosis. Am J Med.

[2] Burge RT, Worley D, Johansen A, Bhattacharyya S, Bose U (2001). The cost of osteoporotic fractures in the UK: projections for 2000–2020. J Med Econ.

[3] Foundation NO (2018). What is Osteoporosis? National osteoporosis foundation website.

[4] Collins FL, Rios-Arce ND, Schepper JD, Parameswaran N, McCabe LR (2017). The potential of probiotics as a therapy for osteoporosis. Microbiol Spectr.

[5] Manolagas SC (2010). From estrogen-centric to aging and oxidative stress: a revised perspective of the pathogenesis of osteoporosis. Endocr Rev.

[6] Malutan AM, Dan M, Nicolae C, Carmen M (2014). Proinflammatory and anti-inflammatory cytokine changes related to menopause. Menopausal Rev.

[7] D’Amelio Patrizia, Grimaldi Anastasia, Pescarmona Gian Piero, Tamone Cristina, Roato Ilaria, Isaia Giancarlo (2005). Spontaneous osteoclast formation from peripheral blood mononuclear cells in postmenopausal osteoporosis. The FASEB Journal.

[8] D’Amelio P, Grimaldi A, Di Bella S, Brianza SZM, Cristofaro MA, Tamone C (2008). Estrogen deficiency increases osteoclastogenesis up-regulating T cells activity: a key mechanism in osteoporosis. Bone.

[9] Pacifici R, Brown C, Puscheck E, Friedrich E, Slatopolsky E, Maggio D (1991). Effect of surgical menopause and estrogen replacement on cytokine release from human blood mononuclear cells. Proc Natl Acad Sci U S A.

[10] Bismar H, Diel I, Ziegler R, Pfeilschifter J (1995). Increased cytokine secretion by human bone marrow cells after menopause or discontinuation of estrogen replacement. J Clin Endocrinol Metab.

[11] Passeri G, Girasole G, Jilka RL, Manolagas SC (1993). Increased interleukin-6 production by murine bone marrow and bone cells after estrogen withdrawal. Endocrinology.

[12] Bossen C, Ingold K, Tardivel A, Bodmer J-L, Gaide O, Hertig S (2006). Interactions of tumor necrosis factor (TNF) and TNF receptor family members in the mouse and human. J Biol Chem.

[13] Bamias G, Mishina M, Nyce M, Ross WG, Kollias G, Rivera-Nieves J (2006). Role of TL1A and its receptor DR3 in two models of chronic murine ileitis. Proc Natl Acad Sci U S A.

[14] Collins Fraser L., Williams Jessica O., Bloom Anja C., Singh Ravinder K., Jordan Lauren, Stone Michael D., McCabe Laura R., Wang Eddie C.Y., Williams Anwen S. (2017). CCL3 and MMP-9 are induced by TL1A during death receptor 3 (TNFRSF25)-dependent osteoclast function and systemic bone loss. Bone.

[15] Harris L, Senagore P, Young VB, McCabe LR (2009). Inflammatory bowel disease causes reversible suppression of osteoblast and chondrocyte function in mice. Am J Physiol Gastrointest Liver Physiol.

[16] Meylan F, Richard AC, Siegel RM (2011). TL1A and DR3, a TNF family ligand-receptor pair that promotes lymphocyte costimulation, mucosal hyperplasia, and autoimmune inflammation. Immunol Rev.

[17] Jones GW, Stumhofer JS, Foster T, Twohig JP, Hertzog P, Topley N (2011). Naive and activated T cells display differential responsiveness to TL1A that affects Th17 generation, maintenance, and proliferation. FASEB J.

[18] Collins FL, Williams JO, Bloom AC, Stone MD, Choy E, ECY W (2015). Death receptor 3 (TNFRSF25) increases mineral apposition by osteoblasts and region specific new bone formation in the axial skeleton of male DBA/1 mice. J Immunol Res.

[19] Bull MJ, Williams AS, Mecklenburgh Z, Calder CJ, Twohig JP, Elford C (2008). The death receptor 3-TNF-like protein 1A pathway drives adverse bone pathology in inflammatory arthritis. J Exp Med.

[20] Kotani M, Kikuta J, Klauschen F, Chino T, Kobayashi Y, Yasuda H (2013). Systemic circulation and bone recruitment of osteoclast precursors tracked by using fluorescent imaging techniques. J Immunol.

[21] Borysenko CW, García-Palacios V, Griswold RD, Li Y, Iyer AKV, Yaroslavskiy BB (2006). Death receptor-3 mediates apoptosis in human osteoblasts under narrowly regulated conditions. J Cell Physiol.

[22] Pacifici R (2012). Role of T cells in ovariectomy induced bone loss--revisited. J Bone Miner Res.

[23] Bamias G, Siakavellas SI, Stamatelopoulos KS, Chryssochoou E, Papamichael C, Sfikakis PP (2008). Circulating levels of TNF-like cytokine 1A (TL1A) and its decoy receptor 3 (DcR3) in rheumatoid arthritis. Clin Immunol.

[24] Clarke BL, Khosla S (2010). Physiology of bone loss. Radiol Clin North Am.

[25] Slebioda TJ, Bojarska-Junak A, Cyman M, Landowski P, Kaminska B, Celinski K (2017). Expression of death receptor 3 on peripheral blood mononuclear cells differes in adult IBD patients and children with newly diagnosed IBD. Cytometry B Clin Cytom.

[26] De la Fuente M, Medina S, Del Rio M, Ferrández MD, Hernanz A (2000). Effect of aging on the modulation of macrophage functions by neuropeptides. Life Sci.

[27] Fietta A, Merlini C, De Bernardi PM, Gandola L, Piccioni PD, Grassi C (1993). Non specific immunity in aged healthy subjects and in patients with chronic bronchitis. Aging (Milano).

[28] Sadeghi HM, Schnelle JF, Thoma JK, Nishanian P, Fahey JL (1999). Phenotypic and functional characteristics of circulating monocytes of elderly persons. Exp Gerontol.

[29] Cenci S, Weitzmann MN, Roggia C, Namba N, Novack D, Woodring J (2000). Estrogen deficiency induces bone loss by enhancing T-cell production of TNF-alpha. J Clin Invest.

[30] Tyagi Abdul M., Srivastava Kamini, Mansoori Mohd Nizam, Trivedi Ritu, Chattopadhyay Naibedya, Singh Divya (2012). Estrogen Deficiency Induces the Differentiation of IL-17 Secreting Th17 Cells: A New Candidate in the Pathogenesis of Osteoporosis. PLoS ONE.

[31] Jin S, Chin J, Seeber S, Niewoehner J, Weiser B, Beaucamp N (2013). TL1A/TNFSF15 directly induces proinflammatory cytokines, including TNFα, from CD3+CD161+ T cells to exacerbate gut inflammation. Mucosal Immunol.

[32] Zhou M, Liu R, Su D, Feng X, Li X (2014). TL1A increased the differentiation of peripheral Th17 in rheumatoid arthritis. Cytokine.

